# Supplementary figures and images for: Performance comparison of Agilent new SureSelect All Exon v8 probes with v7 probes for exome sequencing
Source: BMC Genomics. 2022 Aug 12;23:582. doi: 10.1186/s12864-022-08825-w (PMC9375261; doi:10.1186/s12864-022-08825-w)

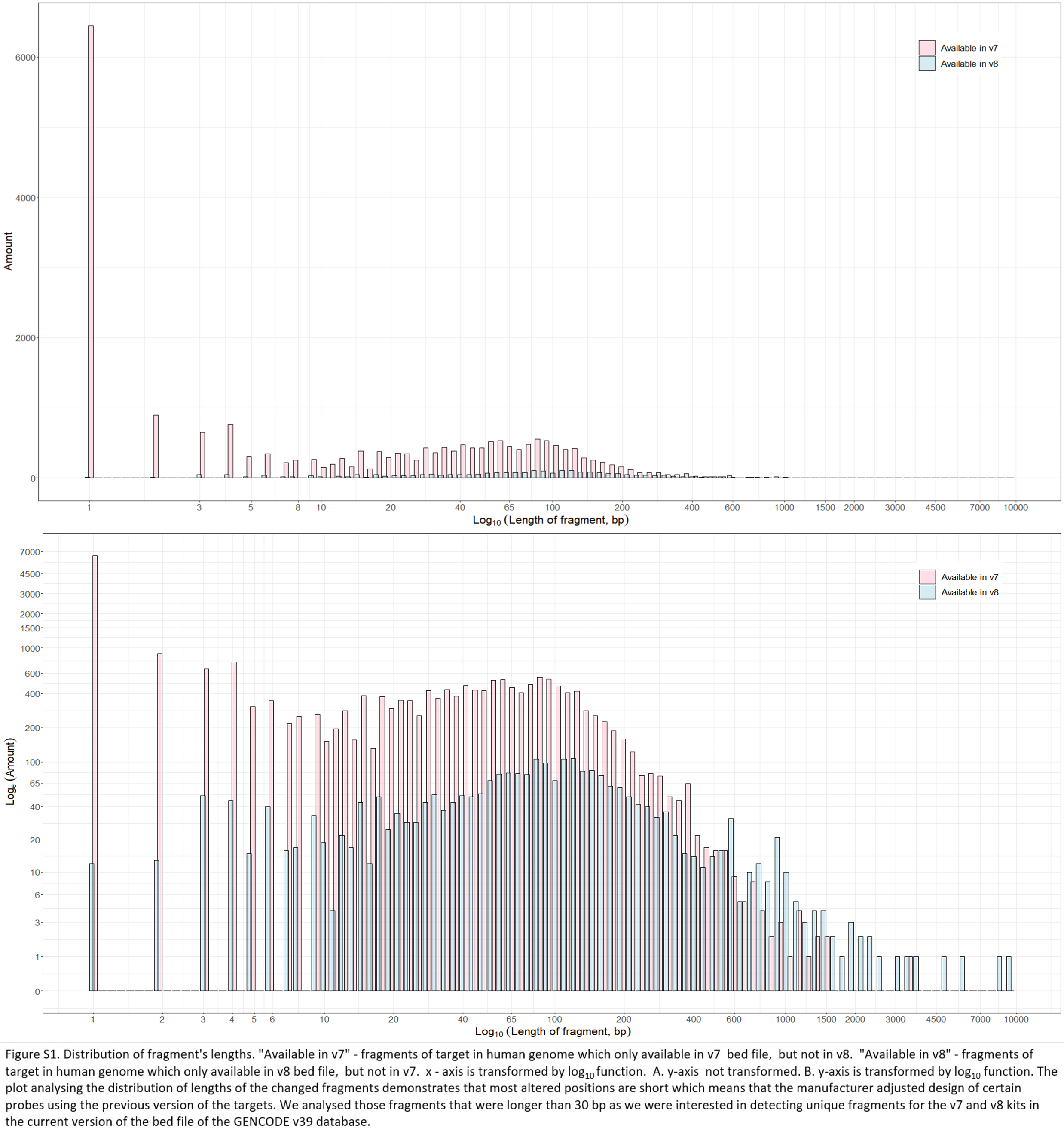

Supplement: Supplementary file 4 — Additional file 4: Figure S1. Distribution of fragments lengths. [file 12864_2022_8825_MOESM4_ESM.png]
